# Actin Stabilization by Jasplakinolide Affects the Function of Bone Marrow-Derived Late Endothelial Progenitor Cells

**DOI:** 10.1371/journal.pone.0050899

**Published:** 2012-11-30

**Authors:** Xiaoyun Zhang, Xiaodong Cui, Lixia Cheng, Xiumei Guan, Hong Li, Xin Li, Min Cheng

**Affiliations:** 1 Medicine Research Center, Weifang Medical College, Weifang, Shandong, People’s Republic of China; 2 Department of Endocrinology, People’s Hospital, Weifang, Shandong, People’s Republic of China; INSERM, France

## Abstract

**Background:**

Bone marrow-derived endothelial progenitor cells (EPCs), especially late EPCs, play a critical role in endothelial maintenance and repair, and postnatal vasculogenesis. Although the actin cytoskeleton has been considered as a modulator that controls the function and modulation of stem cells, its role in the function of EPCs, and in particular late EPCs, remains poorly understood.

**Methodology/Principal Finding:**

Bone marrow-derived late EPCs were treated with jasplakinolide, a compound that stabilizes actin filaments. Cell apoptosis, proliferation, adhesion, migration, tube formation, nitric oxide (NO) production and endothelial NO synthase (eNOS) phosphorylation were subsequently assayed in vitro. Moreover, EPCs were locally infused into freshly balloon-injured carotid arteries, and the reendothelialization capacity was evaluated after 14 days. Jasplakinolide affected the actin distribution of late EPCs in a concentration and time dependent manner, and a moderate concentration of (100 nmol/l) jasplakinolide directly stabilized the actin filament of late EPCs. Actin stabilization by jasplakinolide enhanced the late EPC apoptosis induced by VEGF deprivation, and significantly impaired late EPC proliferation, adhesion, migration and tube formation. Furthermore, jasplakinolide attenuated the reendothelialization capacity of transplanted EPCs in the injured arterial segment in vivo. However, eNOS phosphorylation and NO production were increased in late EPCs treated with jasplakinolide. NO donor sodium nitroprusside (SNP) rescued the functional activities of jasplakinolide-stressed late EPCs while the endothelial NO synthase inhibitor L-NAME led to a further dysfunction induced by jasplakinolide in late EPCs.

**Conclusions/Significance:**

A moderate concentration of jasplakinolide results in an accumulation of actin filaments, enhancing the apoptosis induced by cytokine deprivation, and impairing the proliferation and function of late EPCs both in vitro and in vivo. NO donor reverses these impairments, suggesting the role of NO-related mechanisms in jasplakinolide-induced EPC downregulation. Actin cytoskeleton may thus play a pivotal role in regulating late EPC function.

## Introduction

Loss of endothelial integrity and impaired capacity for neovascularization are thought to contribute to cardiovascular diseases, such as atherosclerosis, ischemic events in limbs, retina and myocardium [1], . Recent studies have shown that endogenous re-endothelialization and postnatal neovascularization rely not only on the migration, proliferation and sprouting of preexisting mature endothelial cells, but also on the activity of EPCs [3], [4]. EPCs possess the capability to interact with the endothelial layer of different organs in a way that causes morphological changes and strong adhesion to the tissue [5]. They promote reendothelialization or stimulate angiogenesis directly by the differentiation into mature endothelial cells and also indirectly by their secretary factors that mobilize endothelial and progenitor cells to take part in angiogenesis and reconstruction [6]. Since dysfunction or decrease in EPCs is linked with high cardiovascular risk, EPCs have been employed as a potential therapeutic means in vascular disorders [7].

Recent studies have demonstrated that EPCs are actually a heterogeneous population and can, according to their morphology, function and growth potential, be dissected into early and late EPCs. The early EPCs appear within 4 to 7 days of culture, are spindle-shaped, and have a limited proliferation potential. The late EPCs develop after 2 to 3 weeks of culture and have the characteristic of endothelium lineage, with a cobblestone shape and long-term proliferation and clonogenic potentials. Moreover, late EPCs show typical endothelial markers, such as vWF, VEGFR-2, VE-cadherin and PECAM-1, but are negative for CD45. Furthermore, like mature endothelial cells, these cells can form the branched tubular structures on extracellular matrix in vitro and new blood vessels or become a part of the systemic circulation system in vivo [8]–[13]. Despite favorable in vitro and vivo angiogenic properties compared with other putative EPCs, late EPCs have been much less studied.

The actin cytoskeleton is accountable for a variety of cell physiological events, such as the formation of stress fibers, adhesion, migration, apoptosis and receptor clustering in different cell models [14], [15]. In recent years, with further developments in stem cell research, the actin cytoskeleton has been considered as a novel modulator that controls the function and modulation of stem cells [16], [17]. However, its role in the function of EPCs, especially late EPCs, remains poorly understood.

To study the possibility that the actin cytoskeleton is involved in the function of late EPCs, cells were treated with the actin-binding cyclodepsipeptide jasplakinolide that stabilizes actin microfilaments and promotes actin polymerization in vitro [18]. The various functions of late EPCs both in vitro and in vivo, including apoptosis, proliferation, adhesion, migration, in vitro tube formation and in vivo reendothelialization capacity were then evaluated using a variety of experimental tools.

## Results

### Characterization of Bone Marrow-derived Late EPCs

**Figure 1:**
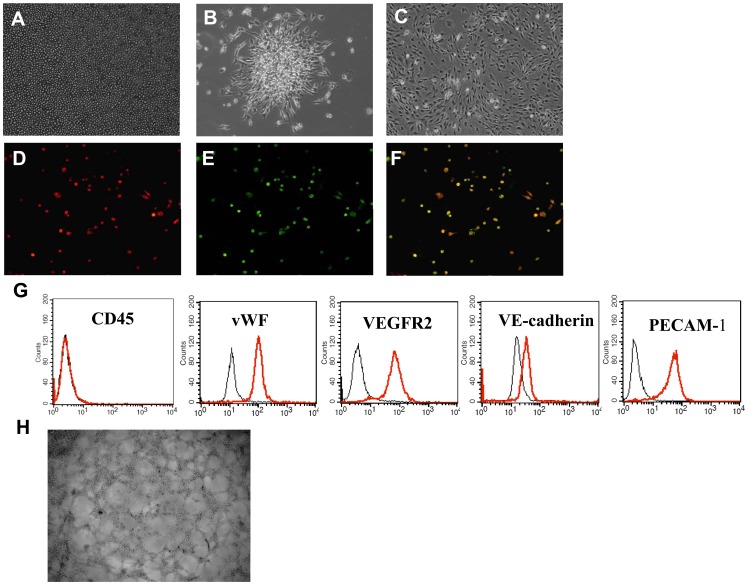
Characterization of late EPCs derived from bone marrow. A: MNCs were isolated and plated on fibronectin-coated culture dish on the first day (100×). B: Seven days after plating (100×). C: After 3–4 weeks, the third-fifth passage cells, namely late EPCs (100×). D-F: DiI-acLDL (red) and fluorescein isothiocyanate UEA-1(green) staining (100×). G: Flow cytometry analysis using several markers. H: Representative image of tubuli like structures formed on Matrigel by late EPCs (40×).

The bone marrow-derived MNCs that initially seeded were round (Fig. 1A). After 7 days, the colonies appeared with the round cells in the centers and the typical spindle cells at the peripheries (Fig. 1B). Late EPCs appeared after 3–4 weeks and showed characteristic homogeneity and cobblestone-like morphology similar to mature endothelial cells (Fig. 1C). The cells were identified as double-positive for Dil-acLDL uptake and lectin binding affinity (Fig. 1D, E, F). FACS analysis revealed these cells did not express CD45 but the majority of the cells expressed endothelial-specific markers, such as vWF, VEGFR-2, VE-cadherin and PECAM-1 (Fig. 1G). Moreover, late EPCs successfully formed tubuli like structure on Matrigel (Fig. 1H).

### Concentration- and Time-dependent Effects of Jasplakinolide on the Actin Distribution of Late EPCs

**Figure 2:**
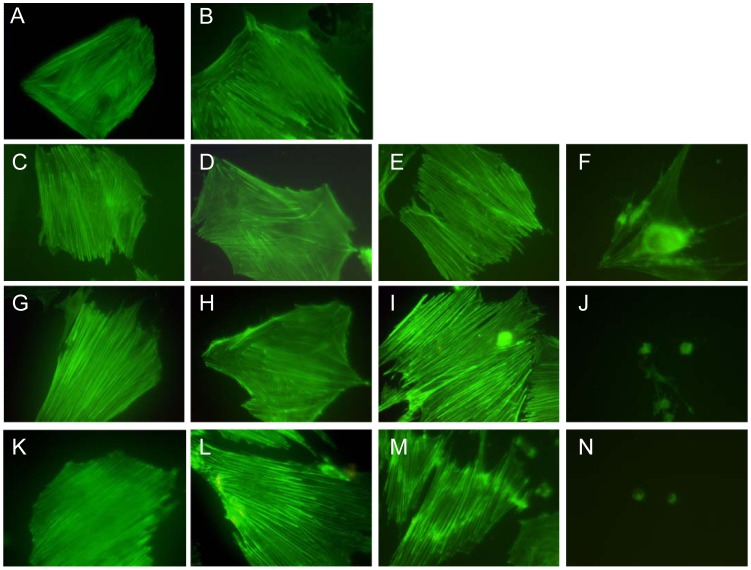
Dose- and time-dependent effects of jasplakinolide on actin distribution of late EPCs. The actin filaments of late EPCs were stained with FITC-phalloidin. (A) Control cells. (B) Cells treated with DMSO for 24 h. (C-N) Effects of jasplakinolide on late EPCs for: 50 nmol/L for 15 min, 30 min, 1 h and 24 h respectively (C-F); 100 nmol/L for 15 min, 30 min, 1 h and 24 h respectively (G-J) and 200 nmol/L for 15 min, 30 min, 1 h and 24 h respectively (K-N).

For evaluating the effects of jasplakinolide on the actin distribution of late EPCs, tests were performed using different concentrations of jasplakinolide with various exposure times. The distribution of F-actin in untreated late EPCs exhibits a dense network of parallel stress fibers (Fig. 2A). When treated with 50 nmol/l jasplakinolide for a short time (15 min), no obvious alteration of the actin cytoskeleton was observed (Fig. 2C). With prolonged incubation (24 h), although stress fibers were still visible, the actin masses were found to be aggregated around the peri-nuclear region (Fig. 2F). However, the actin cytoskeleton of late EPCs treated by vehicle DMSO did not show such an alteration (Fig. 2B). Raising the jasplakinolide concentration to 100 nmol/l slightly enhanced the cortical actin filaments of late EPCs after 30 min of incubation (Fig. 2H), and resulted after 1 h in thick actin bundles and a patchy appearance in the cytoplasm (Fig. 2I). After 24 h of incubation, cells contracted, no cortical actin filaments persisted, actin bundles were invisible in the cytoplasm, and only small F-actin masses remained in the peri-nuclear region (Fig. 2J). When a high concentration of jasplakinolide (200 nmol/l) was employed, the alteration of the actin cytoskeleton of late EPCs was seen to be rapid. The cortical actin filaments were now increased after 15 min of treatment only, and the patchy appearance in the cytoplasm was visible after 30 min (Fig. 2K, L). Similarly, F-actin was almost invisible in the cytoplasm, and was replaced by small F-actin masses that were located in the peri-nuclear region after 24 h of incubation (Fig. 2N).

### Stabilization of Actin by Jasplakinolide Enhanced Late EPC Apoptosis Induced by VEGF Deprivation

**Figure 3:**
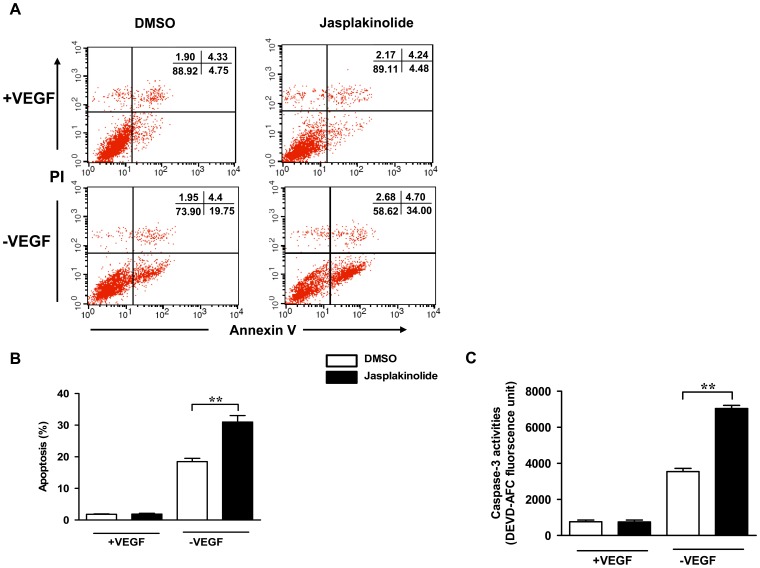
Jasplakinolide enhanced late EPC apoptosis induced by VEGF deprivation. (A) Late EPCs were incubated in the presence or absence of VEGF with jasplakinolide or DMSO for 1 h. The cells were then washed to remove the jasplakinolide or DMSO, and cultured in EGM-2 with or without VEGF. Cells were harvested after 12 h and the apoptotic cells were quantified by FACS after Annexin V FITC and PI staining. Annexin V-positive and PI-negative cells were defined as apoptotic. (B) The proportion of apoptotic cells. (C) Late EPCs were incubated in the absence of VEGF with jasplakinolide or DMSO for 1 h. The cells were then washed to remove the jasplakinolide or DMSO, and cultured in EGM-2 without VEGF for 6 h. Lysate of late EPCs was collected, and the intracellular caspase-3 activity was assayed with an Caspase-3 Fluorescent Assay Kit based on the release of free 7-amino-4-trifluoromethyl coumarin (AFC) by the activated caspase-3 cleavage of DEVD-AFC. Data represent the mean±SE of four different experiments. **P<0.01.

Previous reports have suggested that the alteration of the cytoskeletal actin network is a morphological effecter in apoptosis [19]. To determine whether the stabilization of actin might induce the apoptosis, late EPCs were incubated with jasplakinolide (100 nmol/l) or DMSO (0.02%) in regular EGM-2 (containing VEGF) for 1 h. The cells were then washed to remove the jasplakinolide or DMSO, and they were once more cultured in regular EGM-2. The cells were harvested after 12 h and the apoptotic cells were quantified by FACS after Annexin V and PI staining. As shown in Fig. 3A, B jasplakinolide and DMSO treatments resulted in similar percentages of apoptotic late EPCs. However, the percentages of apoptotic late EPCs after VEGF deprivation were increased after the addition of jasplakinolide at a concentration of 100 nmol/l.

We then explored the underlying mechanism behind the jasplakinolide-augmented apoptosis. The members of the caspase protease family, especially caspase-3, play a key role in the initiation of cellular events during the early apoptotic process, and caspase-3 has also been considered as a good marker to indicate apoptosis. Late EPCs cells were incubated either with jasplakinolide (100 nmol/l) or DMSO (0.02%) in the absence or presence of VEGF for 6 h. Caspase-3-like activity was assayed. Jasplakinolide or DMSO-treatment did not activate caspase-3 in late EPCs cultured with VEGF. However caspase-3-like activity was present in both jasplakinolide and DMSO-treated EPCs after 6 h of VEGF deprivation. Futhermore, in the jasplakinolide-treated cells, a higher caspase-3-like activity was observed than those in DMSO-treated cells (Fig. 3C).

### Stabilization of Actin by Jasplakinolide Led to the Inhibition of Late EPC Proliferation

**Figure 4:**
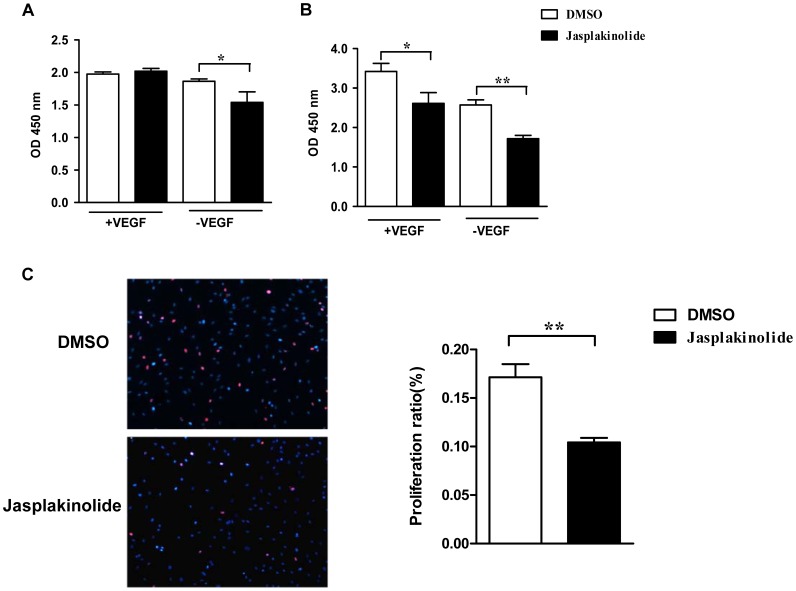
Jasplakinolide decreased late EPC proliferation. (A-B) Late EPCs were incubated in the presence or absence of VEGF with either jasplakinolide or DMSO for 1 h. The cells were then washed to remove the jasplakinolide or DMSO, and cultured in EGM-2 with or without VEGF for 12 (A) or 24 h (B). Cell proliferation was assessed by CCK-8 assay. (C) Late EPCs were incubated in the absence of VEGF with either jasplakinolide or DMSO for 1 h. The cells were then washed to remove the jasplakinolide or DMSO, and cultured in EGM-2 without VEGF for 12 h. Cell proliferation was assessed by the EdU incorporation assay. More than five random fields per well were captured at 200× magnification, and IPP 6.0 was used to calculate the percentage of EdU-positive cells (identified by Apollo® 567 fluorescence) in total cells (identified by Hoechst33342 nuclei staining). Data represent the mean±SE of four different experiments. *P<0.05, **P<0.01.

Late EPCs were incubated in the presence or absence of VEGF with jasplakinolide or with DMSO for 1 h. The cells were then washed to remove the jasplakinolide or DMSO, after which they were cultured in EGM-2 with or without VEGF for further 12 or 24 h. Cell proliferation was assessed by CCK-8 assay. At 12 h, the proliferation activity of late EPCs incubated with jasplakinolide was observed to be similar to that in DMSO-treated cells in the presence of VEGF, but a statistical difference was observed after withdrawal from VEGF (Fig. 4A). At 24 h, jasplakinolide inhibited late EPC proliferation in the presence of VEGF, and VEGF deprivation exacerbated the impaired late EPC proliferation due to jasplakinolide (Fig. 4B). Indeed, the EdU incorporation assay confirmed that the stabilization of actin by jasplakinolide inhibited the proliferation of VEGF deprived EPCs (Fig. 4C).

### Jasplakinolide Impaired Late EPC Adhesion by Stabilizing F-actin

**Figure 5:**
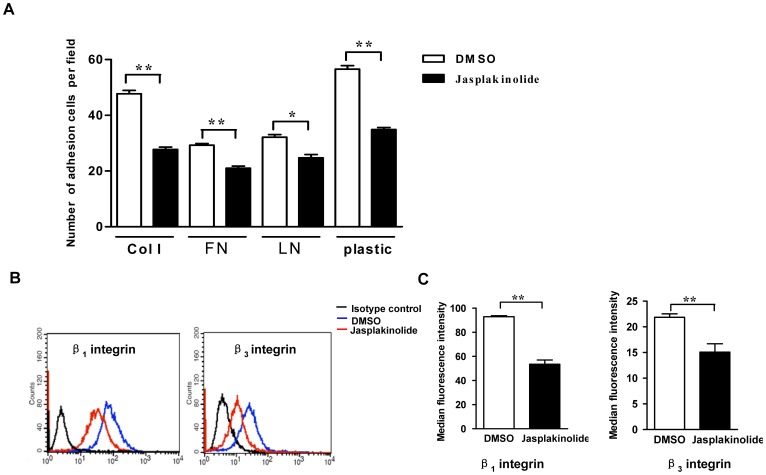
Jasplakinolide inhibited the adhesion of late EPCs. (A) Late EPCs were pretreated with either jasplakinolide or DMSO for 1 h and then seeded on either plastic or culture surfaces coated with different ECM proteins, such as fibronectin, collagen I or laminin, and incubated for 1 h at 37°C. After nonadherent cells were removed by washing, adherent cells were counted and analyzed. (B) The cell surface expressions of integrin β1 and β3 were assessed by FACS. Representative FACS profiles of four independent experiments are shown. The relative fluorescence intensity that is normalized with the mean fluorescence intensity of isotype control. Data represent the mean±SE of four different experiments. **P<0.01.

A recent study has reported that remodeling the actin cytoskeleton can influence the ability of cell adhesion [20]. To test the effects of the actin rearrangement induced by jasplakinolide on late EPC adhesiveness, late EPCs were pretreated with jasplakinolide 100 nmol/l or DMSO (0.02%) for 1 h and then seeded on either plastic or culture surfaces coated with different ECM proteins, such as fibronectin (FN), collagen I (Col I) and laminin (LN). After incubation for 1 h at 37°C, late EPC adhesion was observed under microscope. As shown in Fig. 5A, the stabilization of actin by jasplakinolide impaired the EPC adhesion both on plastic and on ECM proteins compared with that in DMSO-treated cells. As integrins β1 and β3 affect the EPC adhesion [8], we next measured the expressions of these integrins on the late EPC surface by flow cytometry. The results show that the expressions of integrins β1 and β3, and in particular that of β1 integrin, were significantly decreased by the treatment with jasplakinolide (Fig. 5B, C).

### Stabilization of Actin by Jasplakinolide in Late EPCs Inhibited Migration

**Figure 6:**
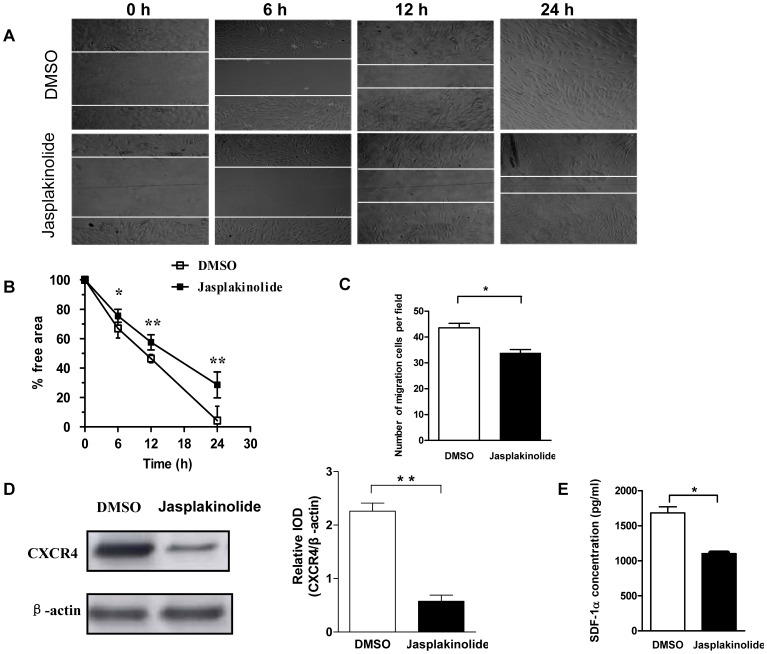
Jasplakinolide impaired late EPC spontaneous migration. (A) Wound was scraped across monolayer late EPCs using a sterile 10 µl pipette tip, and the healing of the scratch wound was monitored at different time points (0, 6, 12 and 24 h) by phase-contrasted microscopy. (B) The results were examined in a blind fashion and determined using an automatic software-assisted image analysis. (C) Spontaneous migration was measured in a modified Boyden chamber assay. Cultured late EPCs were treated with jasplakinolide or DMSO for 1 h. Cells (1×105 cells) were placed in the upper chamber, and the lower chamber was filled with medium and incubated for 16 h. The migrated cells were stained with DAPI and analyzed. (D) Cell lysates were resolved on 12% SDS-PAGE, followed by transfer to PVDF membrane. Western blot was carried out with specific antibody for CXCR4, and each band was detected by the ECL reagent. In addition, the β-actin was analyzed as loading control. (E) Cultured late EPCs were treated with jasplakinolide or DMSO. The supernatants were collected, and the content of SDF-1 was analyzed by ELISA. Data represent the mean±SE of four different experiments. *P<0.05, **P<0.01.

Cell spontaneous migration is tightly coupled to actin assembly [21]. Therefore, we tested whether the jasplakinolide treatment affects late EPC migration. To this end, confluent cells were subjected to jasplakinolide or DMSO for 1 h and a scrape wound was generated. Images were captured at the beginning, and at subsequent regular intervals. A significantly decreased closure of the cell free area was seen as a consequence of the jasplakinolide treatment (Fig. 6A, B). As the repairing of the scratch depends on not only cell migration but also proliferation, we also determined the spontaneous migration of late EPCs by Boyden chamber assay. Late EPCs were cultured in the presence of jasplakinolide or DMSO for 1 h before being placed in the upper compartment of a Boyden chamber, the lower compartment of which contained the same medium as the upper chamber. It was seen that jasplakinolide impairs late EPC migration through the membrane, with 23% less cells passing through as compared with the DMSO treated cells (Fig. 6C). To further understand the underlying mechanism of the effects of jasplakinolide on the migration, we explored the expression of the CXCR4 and SDF-1 systems. The results show the decrease in the CXCR-4 and SDF-1 protein expressions of jasplakinolide-treated late EPCs (Fig. 6 D, E).

### Late EPCs Treated with Jasplakinolide Displayed Impaired Tube Formation Ability

**Figure 7:**
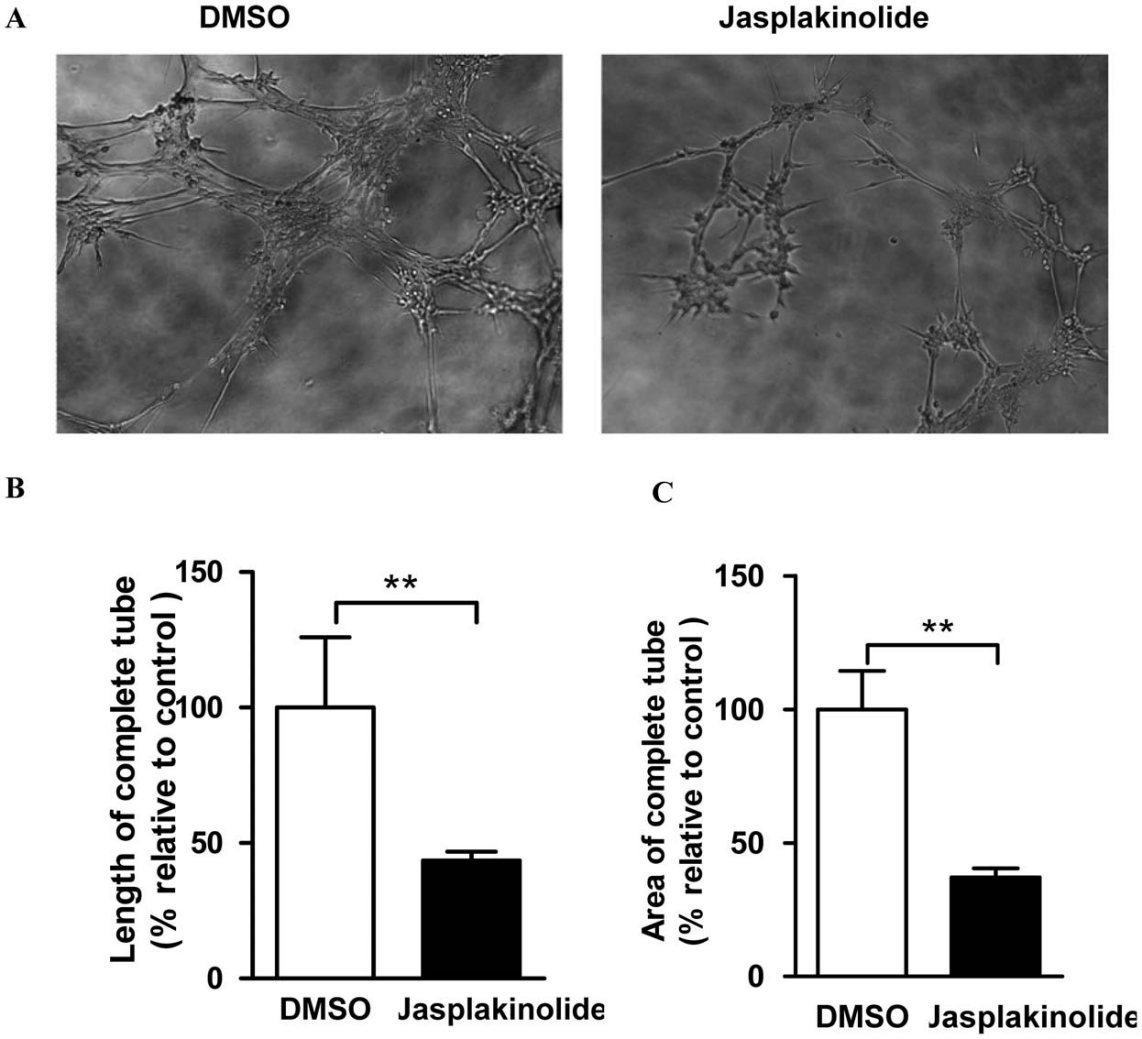
late EPCs treated with jasplakinolide displayed impaired tube formation ability. (A) Representative images of capillary networks formed by late EPCs treated with jasplakinolide or DMSO. The averages of the length (B) and total area (C) of complete tubes formed by cells were compared by computer software. Data represent the fold increase by comparison to the untreated cells (arbitrarily = 100). **P<0.01.

Vascular tube formation results from several mechanisms, including cell proliferation and migration. Given our observation that jasplakinolide enhances apoptosis, and decreases proliferation, adhesion and migration, we further tested using Matrigel assays whether it also diminishes tube formation of late EPCs. Late EPCs pre-treated with DMSO, but not jasplakinolide, generated well-defined tubule-like structures (Fig. 7A). Quantitative assessment of tube formation was conducted by measuring the length and area of closed capillary tube networks. A reduction in tube formation was observed in late EPCs cultured under jasplakinolide conditions compared with DMSO treated EPCs (Fig. 7B, C).

### Jasplakinolide Impaired in vivo Reendothelialization Capacity of Late EPCs

**Figure 8:**
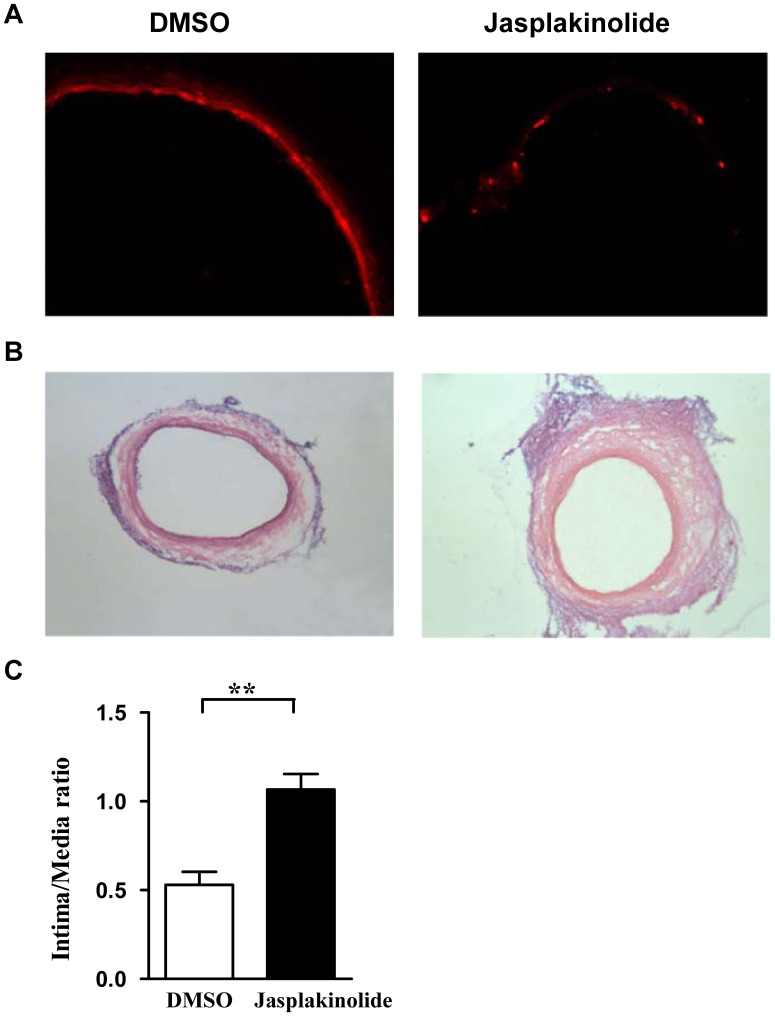
Jasplakinolide impaired in vivo reendothelialization capacity of late EPCs. (A) Cultured late EPCs were treated with jasplakinolide or DMSO for 1 h and then labeled with CM-Dil (Invitrogen, USA). EPCs (1×106) were locally infused into freshly balloon-injured carotid arteries in rat. After 14 days, a fluorescent microscope was performed to detect transplanted EPCs to the sites of vascular injury. (B) Vessels were perfusion-fixed with 10% buffered formalin solution 14 days after endovascular injury and EPC seeding. Representative photomicrographs of hematoxylin-eosin-stained carotid arteries. Original magnification ×40. (C) Hematoxylin-eosin-stained cross-sections were analyzed for neointimal thickening. Intima area/media area ratio were evaluated by computer-assisted histomorphometry. Data represent the fold increase by comparison to the untreated cells (arbitrarily = 100). **P<0.01.

To verify whether the down-regulated function induced by jasplakinolide in late EPCs in vitro affects in vivo reendothelialization, late EPCs treated with jasplakinolide or DMSO were locally infused into freshly balloon-injured carotid arteries. After 14 days, fluorescent microscope revealed that transplanted EPCs were located at the sites of injured arterial. Unlike for the DMSO treated EPCs, jasplakinolide treated EPCs had not formed a monolayer on the luminal surface (Fig. 8A). Moreover, EPCs treated with jasplakinolide showed less reendothelialization and more neointimal thickening (Fig. 8B, C).

### Role of NO Signaling in Jasplakinolide Down-regulated EPCs

**Figure 9:**
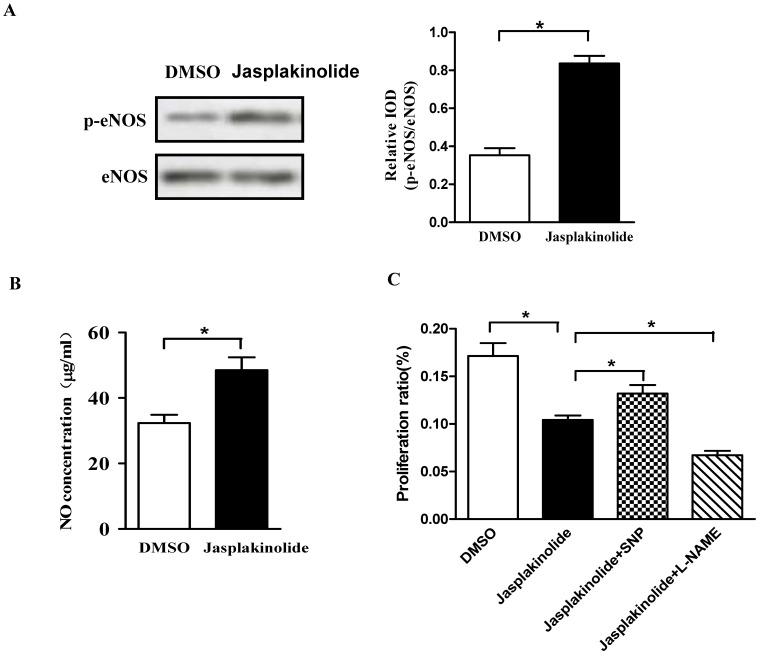
Role of NO signaling in jasplakinolide down-regulated EPCs. (A) After incubation of late EPCs with jasplakinolide or DMSO for 1 h. Phosphorylation of eNOS and total eNOS were assessed by western blot. (B) NO in culture medium was measured by ELISA. (C) Late EPC proliferation was analyzed by the EdU incorporation assay after culture in DMSO or jasplakinolide medium without VEGF in the absence or presence of SNP (NO donor, 25 µmol/l) and L-NAME (NOS inhibitor, 100 µmol/l). Data represent the mean±SE of four different experiments. *P<0.05.

It has been shown that NO is critical to the regulation of EPC function [22]. To investigate the effects of jasplakinolide on NO production in late EPCs, cells were pretreated with jasplakinolide or DMSO, and the protein and cultured media were collected. eNOS phosphorylation and bioavailable NO was assessed by western blot and ELISA, respectively. It was observed that the eNOS phosphorylation was significantly increased in jasplakinolide-treated late EPCs as compared with that in DMSO-treated cells (Fig. 9A). This enhancement in eNOS phosphorylation was associated with a signification release of NO into the cell culture supernatant of late EPCs (Fig. 9B). Moreover, the potential roles of NO–related mechanisms were examined. Coincubation with NO donor SNP significantly ameliorated the inhibitory effect of jasplakinolide on EPC proliferation. In contrast, coincubation with NOS inhibitor l-Ng-nitro-l-arginine methyl ester (L-NAME) significantly enhanced the inhibitory effect of jasplakinolide on EPC number/proliferation (Fig. 9C). These data indicate that jasplakinolide might down-regulate EPCs by modulating NO-related mechanisms.

## Discussion

In the present study, we have demonstrated the biological effects of the stabilization of the actin cytoskeleton by jasplakinolide on late EPCs. The first observation was that jasplakinolide effectively modified the actin cytoskeleton in late EPCs (Fig.2). Moreover, the effects of jasplakinolide on the actin cytoskeleton were concentration and time dependent: at low concentrations of jasplakinolide, the actin organization remained normal, but thick actin bundles appeared around the nucleus. With high concentrations, or long-time incubation, the effects of jasplakinolide on the actin cytoskeleton in late EPCs were disruptive, resulting in the complete disappearance of F-actin. Our findings are consistent with previous studies, e.g. [23]: jasplakinolide has different concentration-dependent effects on the actin cytoskeleton in liver endothelial cells. At a low concentration, such as 50 nmol/l, jasplakinolide only slightly increases the concentration of F-actin in cells. When cells are exposed to moderate concentrations (100∼150 nmol/l) of jasplakinolide, the total mass of F-actin is greatly increased, especially in the peri-nuclear region. At high concentrations of jasplakinolide (200∼300 nmol/l), most of the F-actin bundles disappear and are replaced by diffuse staining, but F-actin clumps are still present. The effects of jasplakinolide at low and moderate concentrations can be explained by its ability to stabilize certain populations of actin filaments, and the reason for the observed disruption at high concentrations most likely is due to jasplakinolide depleting the G-actin, resulting in insufficient polymerization-competent globular actin (G-actin) to maintain normal F-actin turnover [24]. Thus in subsequent experiments, we treated the late EPCs with 100 nmol/l jasplakinolide, which allowed us to investigate a potential role for actin stabilization in regulating the function of late EPCs.

Other major findings from the present study are as follows: 1) Stabilization of actin microfilaments by jasplakinolide augmented the apoptosis of late EPCs deprived of VEGF. 2) Jasplakinolide impaired the functional properties of late EPCs, such as proliferation, adhesion, migration, and in vitro tube formation and in vivo reendothelialization capacity. 3) Jasplakinolide down-regulated the expressions of integrin β1, integrin β3, CXCR4 and SDF-1, but increased eNOS phosphorylation and NO production. 4) NO donor SNP rescued the functional activities of jasplakinolide-stressed late EPCs, but the endothelial NO synthase inhibitor L-NAME led to a further dysfunction induced by jasplakinolide in late EPCs.

Several studies have reported that cytoskeleton alteration induces apoptosis [25]. Furthermore, the key factor in the actin cytoskeleton’s regulation of apoptosis is dynamic redistribution rather than cleavage [26]. One may speculate that actin stabilization by jasplakinolide might result in cell apoptosis. Odaka et al. [27] found the activation of a caspase-3-like protease or proteases was a key in jasplakinolide-induced cell apoptosis. Posey et al. [28]have also demonstrated that jasplakinolide enhances the apoptosis of CTLL-20 cells in response to IL-2 deprivation. In the present study, it was found that jasplakinolide had no effects on the apoptosis of late EPCs in regular culture condition (containing VEGF). This indicated at the concentrations used that jasplakinolide was not toxic to cells, as the percentages of apoptotic late EPCs incubated with VEGF were similar both in the absence and presence of jasplakinolide. As an endothelial cell-specific mitogen and survival factor, VEGF has been shown to exert strong cyto-protective and prosurvival activities [29], [30]. In line with these findings, we found that VEGF deprivation led to the apoptosis of late EPCs, and furthermore that jasplakinolide augmented the apoptosis of late EPCs deprived of VEGF, indicating that the actin stabilization increased the cell sensitivity to cytokine deprivation. Moreover, jasplakinolide appeared to activate the transduction of the apoptotic signal induced by cytokine deprivation, as caspase-3-like activity was increased in jasplakinolide treated, VEGF deprived late EPCs. In the mean time, we also observed that jasplakinolide inhibited the cell proliferation in late EPCs, especially in those late EPCs deprived of VEGF. At 12 h, the proliferation activity of late EPCs incubated with jasplakinolide was similar to DMSO-treated cells in the presence of VEGF. However, withdrawal from VEGF led to a decreased proliferation in jasplakinolide treated late EPCs. At 24 h, jasplakinolide inhibited late EPC proliferation in the presence of VEGF, and VEGF deprivation moreover exacerbated the impaired late EPC proliferation due to jasplakinolide.

It is well accepted that F-actin is at least in part responsible for shaping cell morphology as well as regulating cell spontaneous migration [31]. Several previous studies have shown that cell migration is impaired as a consequence of F-actin aggregate [32], [33]. Stabilization of actin by jasplakinolide significantly inhibited the migration of late EPCs in the present study. Smadja et al [34]have demonstrated that the migration ability of EPCs may be mediated by an autocrine mechanism involving SDF-1/CXCR4. It is thus reasonable to presume that the impaired migration of late EPCs induced by jasplakinolide could also be due to reduced SDF-1/CXCR4 expression. Our data indeed show a reduced protein expression of SDF-1 and CXCR4 in late EPCs treated with jasplakinolide, suggesting that the effect of jasplakinolide on late EPC migration may be also mediated by an autocrine mechanism involving SDF-1/CXCR4.

Actin remodeling plays an essential role in a variety of cellular events, such as cell movement, cytokinesis and cell adhesion [35]. Du et al reported that the actin rearrangement induced by ATP depletion is associated with the loss of cell adhesion [20]. Our data have demonstrated a link between the actin stabilization by jasplakinolide, and the impairment of late EPC adhesion both on plastic and on different ECM proteins, such as fibronectin, collagen I and laminin. Endothelial cell adhesion is mediated by the cell’s surface integrins, specifically α5β1 and αVβ3 [36]. Moreover, late EPCs and endothelial cells have similar expression levels of αVβ3, but late EPCs have a significantly higher level of α5β1 expression compared to endothelial cells [8]. To investigate the mechanism of the impaired adhesion induced by jasplakinolide, the expressions of integrins β1 and β3 on the late EPC surfaces were quantified. Interestingly, we observed that jasplakinolide induced a marked decrease in the protein expressions of integrins β1 and β3, and in particular of integrin β1, on the cell surface, but that it produced no effects on their gene expressions (Figure S2). This indicates that the regulation of jasplakinolide on integrins β1 and β3 is not on the transcriptional level. It is then natural to ask what the possible mechanism underlying the decreased expression of integrins β1 and β3 on the cell surface in jasplakinolide-treated late EPCs is. There is furthermore increasing evidence that the endocytosis of integrins by the cells is accompanied by cytoskeletal reorganization [37], [38]. For example, Schober et al [39]reported that disruption of actin polymerization by cytochalasin E inhibited integrin internalization caused by cellular agonists or anti-LIBS6 in platelet. We therefore suspect that the effects of jasplakinolide on integrins β1 and β3 may be related to internalization. Our preliminary experiments suggest that the integrins β1 and β3 expressions on the cell surfaces of late EPCs incubated with jasplakinolide are higher at 4°C, than at 37°C (data not shown). So internalization might be, at least in part, the reason for the decreased integrins expressions on the late EPC surface since the endocytosis process is temperature dependent [37], [38]. Further work is necessary to confirm this possibility.

It has previously been shown that late EPCs successfully make capillary networks on Matrigel [10]. To better characterize the effects of jasplakinolide on late EPCs, we tested the tubule formation using a Matrigel model. This is a global assay evaluating multiple cellular processes involved in blood vessel growth, such as cell proliferation, migration, adhesion and differentiation. As we have already shown that jasplakinolide inhibits the proliferation, migration and adhesion of late EPCs, it is not surprising that it was also seen to drastically reduce the number of cell extensions formed within the cell networks. In addition, EPCs treated with jasplakinolide showed less reendothelialization and more neointimal thickening in the injured arterial segment in vivo. However, the exact molecular mechanism behind the impairment of the EPC function related to reendothelialization by jasplakinolide is unclear and should be further investigated.

It has been demonstrated that actin cytoskeleton organization is associated with the eNOS expression [40]. Actin stabilization by jasplakinolide leads to a decrease in the binding of the 51-KD ribonucleoprotein to eNOS, increasing the eNOS mRNA expression in endothelial cells [41]. However, mice treated with the actin cytoskeleton disrupter cytochalasin D also show an increased vascular eNOS expression and activity [42]. Moreover, Kolluru et al [43] have shown that depolymerization of the F-actin cytoskeleton induced by hypoxia is associated with low NO availability. Since thus the eNOS expression and activity have been observed in response to both actin-polymerizing and de-polymerizing, they seem to be rather triggered by changes in actin dynamics and/or by cytoskeleton disruption. Here, we have reported that jasplakinolide significantly increases eNOS phosphorylation and bioavaliable NO in late EPCs. Although EPCs express lower levels of eNOS as compared with mature vascular endothelial cells [44], it is nevertheless essential for the survival, migration, and angiogenesis of EPCs [45]. Moreover, NO derived from eNOS has been identified as a critical molecule in mobilizing EPCs from the bone marrow [45], in reducing their senescence [46], and in promoting their proliferation [22]. All of this indicates the important role of NO in maintaining EPC function. One may then naturally ask why the increase of eNOS phosphorylation and NO production is accompanied with impaired late EPC function, such as proliferation, migration and adhesion, in the present study. To answer this, we propose that the increase of eNOS and NO may be related to the intrinsic abilities of the late EPCs to compensate for the impaired function induced by jasplakinolide. Even with this, however, the increase of eNOS phosphorylation and NO production might still not be sufficient to overcome late EPC dysfunction induced by jasplakinolide. Our data further demonstrate that the proliferation of jasplakinolide-stressed late EPCs is rescued after treatment with the NO donor sodium nitroprusside. Moreover, incubation with endothelial NO synthase inhibitor L-NAME aggravates late EPCs dysfunction induced by jasplakinolide.

In conclusion, the results in the present study provide evidence that jasplakinolide exacerbates the apoptosis induced by VEGF deprivation, and impairs the function of late EPCs both in vitro and in vivo. Furthermore, NO–related mechanisms could be the main contributor to jasplakinolide–induced EPC dysfunction. These findings not only indicate that the actin cytoskeleton plays a pivotal role in regulating late EPC function, but also provide further insights into the complex cellular mechanisms of late EPCs in vascular repair.

## Materials and Methods

### Isolation of Bone Marrow Mononuclear Cells and Cell Culture [47]

Whole bone marrow was isolated from both the femurs and tibias of Sprague-Dawley rats (150 to 175 g) (Weifang medical university, China). The bone marrow mononuclear cells were fractionated by density gradient centrifugation (Histopaque®-1083, Sigma, USA) and the leukocyte marker expressions on MNC were evaluated by FACS (Figure S1). MNCs were plated on dishes precoated with fibronectin (Roche, Germany), and were maintained in complete EGM-2 medium (supplemented with EGM-2 bullet kit, including 5% fetal calf serum, recombinant VEGF, recombinant bFGF, Invitrogen, USA). After 4 days in culture, unattached cells were removed by a single washing step with PBS, after which fresh medium was added. Endothelial colonies subsequently appeared (on average 1 colony/107 or 108 plated mononuclear cells). Highly proliferative endothelial cells grew out from these colonies which then formed a confluent monolayer. Cells under passage third-fifth, namely late EPCs, were used for the experiments [8]. This study was carried out in strict accordance with the recommendations in the Guide for the Care and Use of Laboratory Animals of the National Institutes of Health. The protocol was approved by the Committee on the Ethics of Animal Experiments of the Weifang medical college (Permit Number: 5876).

### Identification of Late EPCs

To identify the late EPCs, the cells were characterized by the uptake of 1,1′-dioctadecyl- 3,3,3′,3′-tetramethylindo-carbo-cyanine-labeled acetylated low density lipoprotein (Dil-acLDL, Molecular probes, USA), and by fluorescein isothiocyanate labeled Anti-Ulex Europaeus Lectin 1/UEA1 (FITC-UEA-1, Sigma, USA) staining. In short, the adherent cells were first incubated with 2 µg/ml Dil-acLDL for 1 h, after which they were fixed in 2% paraformaldehyde for 10 min, and then counterstained with 10 µg/ml FITC-UEA-1 for 1 h. After the staining, the samples were viewed with inverted fluorescence microscope (Leica, Germany). Furthermore, the cellular expressions of CD45 (BD, USA, dilution 1∶200), VEGFR2 (eBioscience, USA, dilution 1∶100), von Willebrand factor (Sigma, USA, dilution 1∶200), VE-cadherin (BD, USA, dilution 1∶400) and PECAM-1(eBioscience, USA, dilution 1∶100) were analyzed by fluorescence-activated cell sorter (FACS Calibur, Becton-Dickinson, Palo Alto, CA).

### Fluorescent Staining of Cytoskeleton

Late EPCs were fixed with 4% paraformaldehyde in PBS for 10 min and blocked in PBS containing 1% BSA for 30 min at room temperature. F-actin was stained with FITC-Phalloidin (Enzo Life Sciences, USA) for 45 min, and the images were acquired by using a fluorescence microscope (Leica, Germany).

### FACS Analysis of Apoptosis

Approximately 1×106 late EPCs were double-stained with AnnexinV-FITC and propidium iodide (PI) by using the Annexin V and propidium iodide (PI) apoptosis detection kit (Becton Dickinson, USA) according to the manufacturer’s instructions. Following staining, the cells were washed twice with binding buffer. Apoptotic cells (Annexin V +/PI-) [48]were detected by FACS. Fluorescence parameters were gated using unstained and single-stained cells, and 20,000 cells were counted for each sample. The apoptotic percentage analysis was performed using Cell-Quest software (Becton Dickinson, USA).

### Caspase-3 Activity Assay

The activity of caspase-3 in whole cell lysates was determined using the Caspase-3 Fluorescent Assay Kit (Becton Dickinson, USA) according to the manufacturer’s instructions. Briefly, 1×106 cells were resuspended in 50 µl chilled cell lysis buffer, and incubated on ice for 10 min. Lysates were centrifuged and the supernatants transferred to a new tube with 50 µl 2×reaction buffer/DTT mix. In each reaction, 5 µl of 1 mmol/L caspase-3 substrate (DEVD-AFC) was added to a final concentration of 50 µmol/L. After incubation for 1 h at 37°C, the AFC liberated from the Ac-DEVD-AFC was measured by a spectrofluorometer using 400 nm excitation and measurement of 480–520 nm (peak, 505 nm) emission.

### Cell Proliferation Assay

Cell proliferative activities were examined using CCK-8 (Dojindo, Japan). Briefly, late EPCs were seeded onto 96-well plates (1000 cells/100 µl/well) and the CCK-8 was added to each well according to the manufacturer’s instructions and incubated for 1 h at 37°C. The optical density (OD) value at 450 nm was measured using enzyme-linked immunoabsorbent assay reader (Bio-Rad Laboratories, USA).

Cell proliferation was also examined by Cell-Light™ EdU DNA Cell Proliferation Kit (GuangzhouRibobio Co., Ltd, Guangzhou, China). Late EPCs (1×104 cells/well) were seeded in triplicate in 96-well plates. The EdU (5′-ethynyl-2′-deoxyuridine) incorporation assay was performed according to the manufacturer’s instructions. More than five random fields per well were captured at 200×magnification, and IPP 6.0 was used to calculate the percentage of EdU-positive cells (identified by Apollo® 567 fluorescence) in total cells (identified by Hoechst33342nuclei staining).

### Cell Adhesion Assay

Cells were washed with PBS, and then gently detached with 0.25% trypsin/EDTA. After centrifugation and resuspension with serum-free medium, equal cell numbers were seeded onto either plastic or culture surfaces coated with different ECM proteins, such as fibronectin, collagen I and laminin, and incubated for 1 h at 37°C. After non-adherent cells were removed by washing, adherent cells were counted independently in six random high-power (×100) microscope fields (HPF)/well by three observers unaware of the treatments.

### FACS Analysis of the Expressions of Integrins β1 and β3

The surface expressions of integrins β1 and β3 present on late EPCs were determined by FACS. Cells were trypsinized, incubated with CD29-FITC and CD61-PE antibodies (eBioscience, USA) for 1 h. 20,000 cells were measured for fluorescent intensity per experiment. In addition, isotype controls were performed for each sample condition.

### Wound Healing Assay

Late EPCs were seeded in a 12-well plate until they reached ∼80% cell density. The cell layer was scratched in each well to create a cleared line using a 10 µl pipette tip. It was photographed both before, and at different time points after creating the scratch. The cell-free area was determined using an automatic software-assisted image analysis (Media Cybernetics, USA).

### Modified Boyden Chamber Analysis of the Migration of Late EPCs

The migratory function of late EPCs was evaluated by a modified Boyden chamber (Costar, Cambridge, MA, USA) assay. Briefly, a total of 1×105cells late EPCs were placed in the upper chamber, while the medium without serum and cytokines was placed in the lower chamber. The assays were conducted over a 16 h incubation period at 37°Cin an incubator equilibrated with 5% CO2. The membrane was then washed gently with PBS, and fixed with 4% paraformaldeyde. Non-migrating cells were gently removed with cotton balls from the upper side of the membrane, and the membrane was then stained by using 4′,6-diamidino-2-phenylindole (DAPI). The migration of late EPCs was evaluated by counting the migrated cells in six random high-power (100×) microscope fields/well.

### In vitro Tube Formation on Matrigel Plate

Firstly, a 96-well plate was coated with 100 µl Matrigel (Becton-Dickinson, USA), and incubated at 37°C for 1 h. 2×105 late EPCs/ml were added to each well for 10 h. The enclosed networks of tubes were photographed from six randomly chosen fields under a microscope. The averages of the total number and area of complete tubes formed by late EPCs per unit area were compared by Image-Pro Plus (Media Cybernetics, USA).

### Western Blots Analysis of the Expressions of CXCR4 and Phosphorylated eNOS

Cellular protein was extracted in 150 µl of 1×SDS loading buffer (62.5 mmol/l Tris–HCL pH 6.8, 2% SDS, 10% glycerol, 50 mmol/l DTT, 0.1% bromphenol blue) in the presence of 0.1% EDTA-free protease inhibitor cocktail, 1 mmol/l sodium orthovanadate and 1 mmol/l sodium fluoride. Protein was quantified using the bichoninic acid assay (BCA; Pierce Biotechnology, Rockford, IL) according to the manufacturer’s instructions. Equal amounts of protein (50 µg) were separated through a 12% SDS–PAGE and transferred to a PVDF membrane. Membranes were blocked in 5% milk-TBST, followed by overnight incubation with appropriate primary antibodies against CXCR4 (Abcam), phosphorylated eNOS or eNOS (Chemicon). The β-actin was used as the control measure. Membranes were then washed with TBST and incubated with secondary antibody conjugated to HRP (Santa Cruz). Immunoreactive bands were visualized by chemiluminenscence (Amersham Pharmacia ECL), and the resulting autoradiograms were analyzed by densitometry.

### ELISA Analysis of the Secretion Actions of Late EPCs

To measure the secretion actions of late EPCs, the cultured medium was collected and 10×concentrated by centrifugation for 20 min at 5000×g at 4°C using Ultrafree-4 centrifugal filter tubes with Biomax-5 membrane (Millipore, USA). The levels of SDF-1 and NO in the cell culture supernatants were measured by sandwich enzyme-linked immunosorbent assay (ELISA, RD, USA) according to the manufacturer’s instructions. The optical density (OD) was measured at 450 nm with an enzyme-linked immunoabsorbent assay reader (Bio-Rad Laboratories, USA).

### Animal Model and Cell Transplantation

Balloon injury of the right common carotid artery was performed as described previously [49]. Briefly, rats (350∼400 g) were anesthetized by an intraperitoneal injection of ketamin(100 mg/kg) and xylazine (5 mg/kg), and the right carotid artery was exposed. Surgery was carried out using a dissecting microscope. Hemostatic controls were placed at the proximal common carotid and the internal carotid. A Fogarty 2F embolectomy catheter (Edwards Lifesciences, Unterschleissheim, Germany) was introduced into the external carotid, advanced to the common carotid, inflated, and withdrawn three times with rotation. EPCs (1×106) labeled with CM-Dil (Invitrogen, USA) were suspended in 150 µl PBS supplemented with 20% (v/v) rat serum and heparin (20 U/ml). Cell solution was instilled and incubated in the freshly injured arterial bed for 25∼30 min. Unbound cells were removed by rinsing the isolated arterial segment with PBS. Finally, the catheter was removed, and blood flow was restored. All animal procedures were performed in accordance with institutional guidelines and conformed with the Guide for the Care and Use of Laboratory Animals as published by the US NIH. On day 14 animals were euthanized and perfusion-fixed with 10% buffered formalin. Vessels were embedded in O.C.T. and frozen in liquid nitrogen. For histomorphology cross-sections were stained with hemato xylin/eosin and examined for vessel diameter, media area, and intima to media area ratio. At the same time, exogenously administered CM-Dil-labeled culture EPCs were detected in red fluorescence.

## Supporting Information

Figure S1 **FACS analysis of the MNC fraction after density gradient fractionation.** A: FSC-H ×SSC-H plot. B: Representative histograms on MNC characterized by the expressions of CD45, CD29 and CD34. Plots show isotype controls (black) vs. specific antibody staining (red).
(TIF)

Figure S2 **Effects of jasplakinolide on the mRNA levels of integrins β1 and β3 in late EPCs.** Late EPCs were treated with jasplakinolide or DMSO for 1 h, and mRNAs were measured by real-time quantitative RT-PCR. The results were analyzed with the comparative Ct method (2−ΔΔCt). The data were expressed as an n-fold difference relative to the untreated sample.
(TIF)
