# Supplementary figures and images for: Actin Stabilization by Jasplakinolide Affects the Function of Bone Marrow-Derived Late Endothelial Progenitor Cells
Source: PLoS One. 2012 Nov 30;7(11):e50899. doi: 10.1371/journal.pone.0050899 (PMC3511387; doi:10.1371/journal.pone.0050899)

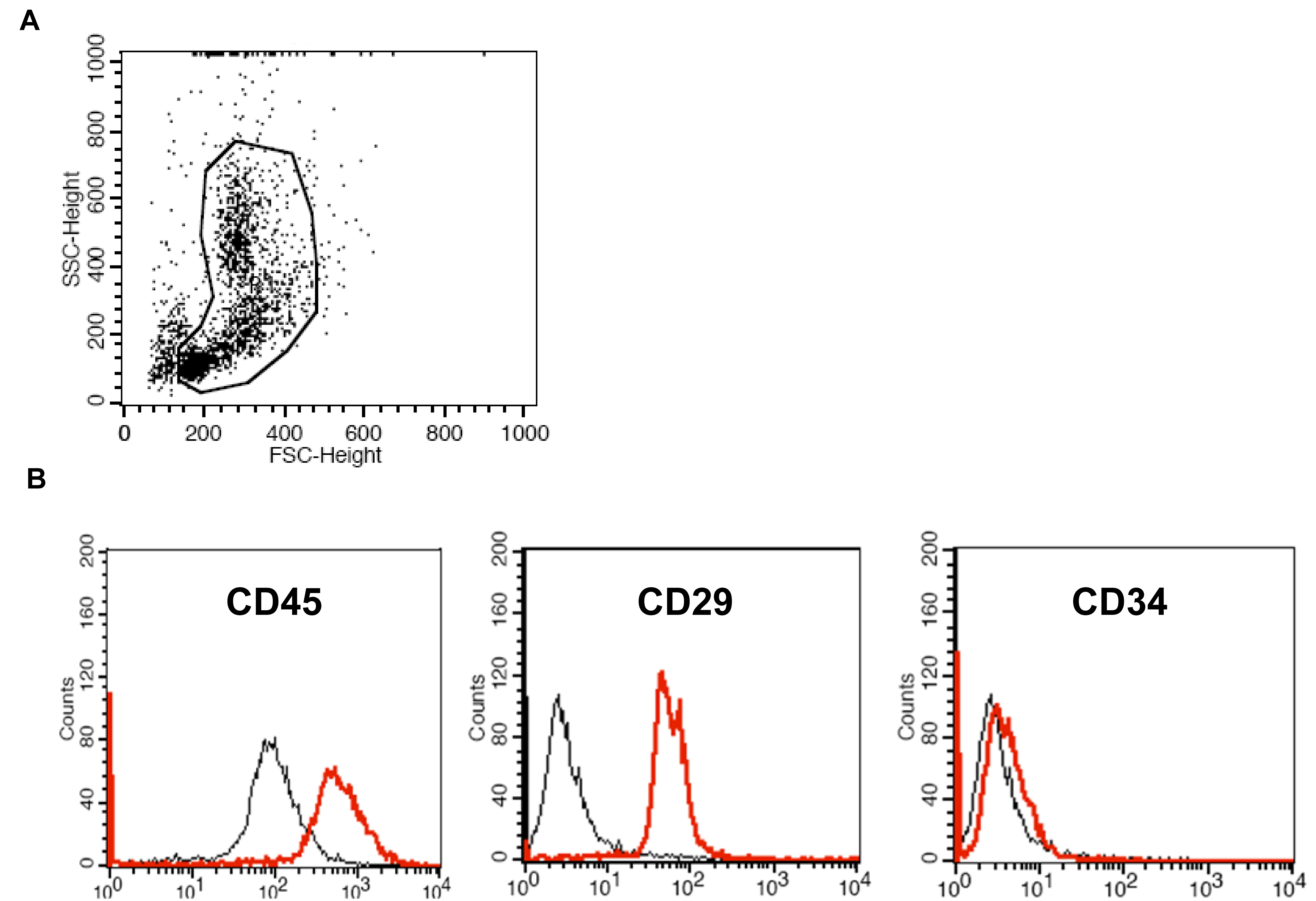

Supplement: Figure S1 — FACS analysis of the MNC fraction after density gradient fractionation. A: FSC-H ×SSC-H plot. B: Representative histograms on MNC characterized by the expressions of CD45, CD29 and CD34. Plots show isotype controls (black) vs. specific antibody staining (red). (TIF) [file pone.0050899.s001.tif]

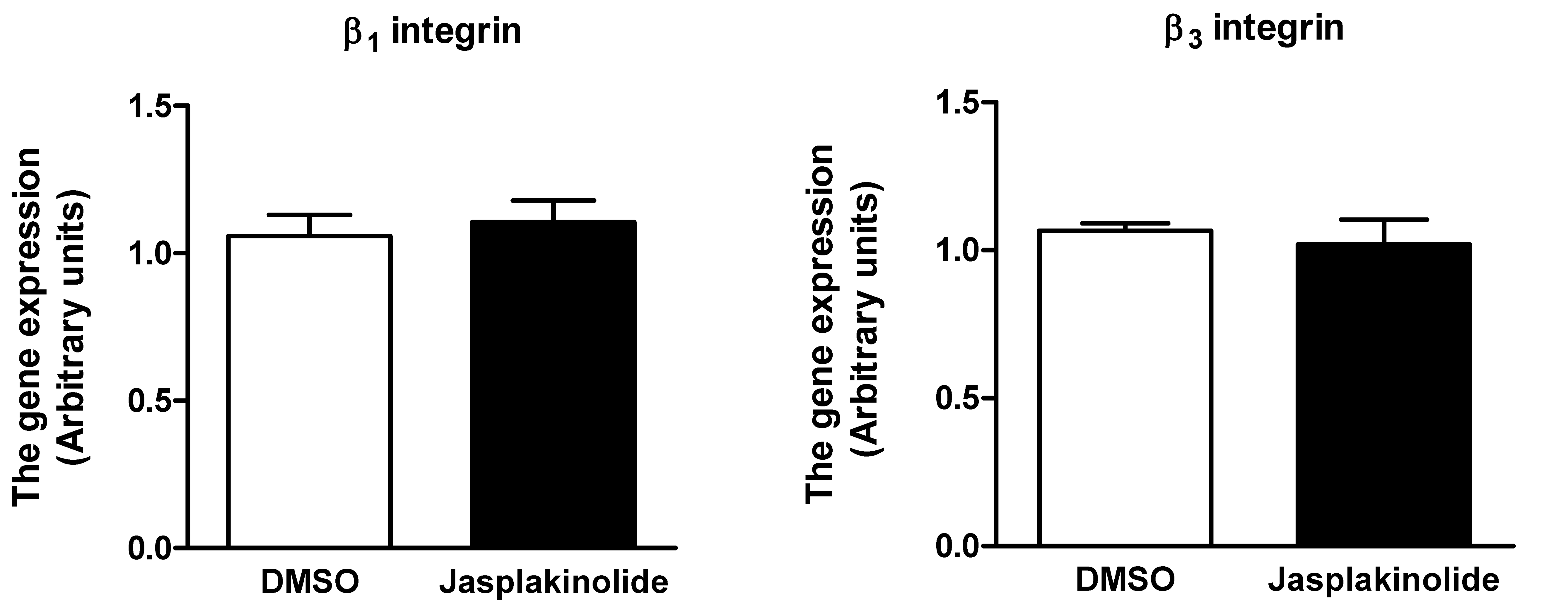

Supplement: Figure S2 — Effects of jasplakinolide on the mRNA levels of integrins β1 and β3 in late EPCs. Late EPCs were treated with jasplakinolide or DMSO for 1 h, and mRNAs were measured by real-time quantitative RT-PCR. The results were analyzed with the comparative Ct method (2−ΔΔCt). The data were expressed as an n-fold difference relative to the untreated sample. (TIF) [file pone.0050899.s002.tif]
